# Letter to the editor: Amyloid‐beta‐targeting monoclonal antibodies for people with mild cognitive impairment or mild dementia due to Alzheimer's disease

**DOI:** 10.1002/14651858.ED000182

**Published:** 2026-10-15

**Authors:** Donatienne Van Weehaeghe, Andrea Varrone, Matthias Brendel, Lisbeth Marner, Antoine Verger, Nelleke Tolboom, Igor Yakushev, Francesco Fraioli, Pablo Aguiar Fernandez, Valentina Berti, Roland Wiest, Federica Agosta, Massimo Filippi

**Affiliations:** Department of Radiology and Nuclear MedicineGhent University HospitalGhentBelgium9000; Department of Clinical NeuroscienceCentre for Psychiatry Research, Karolinska Institutet & Stockholm health care servicesStockholmSweden171 77; Department of Nuclear MedicineLMU University Hospital, LMU MunichMunichGermany; Department of Nuclear MedicineCopenhagen University Hospital Herlev and GentofteCopenhagenDenmark; Department of Nuclear Medicine and Nancyclotep Imaging PlatformIADI, INSERM U1254, Université de LorraineNancyFrance; Department of Radiology and Nuclear MedicineUniversity Medical Centre UtrechtUtrechtThe Netherlands; Department of Nuclear MedicineSchool of Medicine, TUM University Hospital, Technical University of MunichMunichGermany; Institute of Nuclear MedicineUniversity College LondonLondonUnited Kingdom; CIMUS, Universidade Santiago de Compostela & Nuclear Medicine DepartmentUniversity Hospital IDIS, Santiago de CompostelaSpain; Nuclear Medicine Unit, Department of Experimental and Clinical Biomedical SciencesUniversity of FlorenceItaly; Institute of Diagnostic and Interventional NeuroradiologyInselspital, Bern University HospitalBernSwitzerland; Center for Alzheimer's disease and related disorders (CARD)Unit of Neurology, IRCCS Ospedale San Raffaele and Vita-Salute San Raffaele UniversityMilanItaly; Center for Alzheimer's disease and related disorders (CARD)Unit of Neurology, IRCCS Ospedale San Raffaele and Vita-Salute San Raffaele UniversityMilanItaly


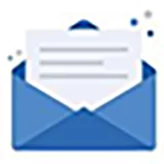


### Declarations of interest

***PAF, AV, FF, NT****and* ***VB****declare no competing interests.* ***LM****declares employment as a hospital clinician in the Capital Region of Denmark.* ***AV****declares membership of the GE Healthcare advisory board.* ***IY****declares speaker honoraria from Piramal and Life Molecular Imaging and involvement in studies eligible for inclusion in the Cochrane review.* ***MB****declares speaker and advisory fees from GE Healthcare, Life Molecular Imaging and IBA.* ***RW****declares speaker-related support from Eli Lilly.* ***FA****declares payments for speaker, consultancy, honorarium or advisory activities from Biogen Idec, Bristol Myers Squibb, Eisai, Eli Lilly, GE Healthcare, Neuraxpharm and Roche, and involvement in studies eligible for inclusion in the Cochrane review.* ***MF****declares payments and advisory or expert roles involving Biogen, Bristol Myers Squibb, Eisai, Eli Lilly, GE Healthcare and Roche, and involvement in studies eligible for inclusion in the Cochrane review.* ***DVW****declares advisory board membership for Eli Lilly, speaker honoraria from GE Healthcare, reader honoraria from Life Molecular Imaging, and research support from GE Healthcare.*

This letter is sent on behalf of the European Association of Nuclear Medicine. A recent meta‐analysis by Nonino et al concluded that successful removal of cerebral amyloid is not associated with clinically meaningful results in people with mild cognitive impairment or mild dementia due to Alzheimer's disease (AD) [Bibr ED000182-bib-0001]. According to the authors amyloid‐beta(Aβ)‐targeting monoclonal antibodies are only associated with adverse events by increasing the risk of amyloid‐related imaging abnormalities with only trivial effect on cognitive function, dementia severity at 18 months or functional outcome. Nevertheless, several limitations of this meta‐analysis should be considered, in line with the recent paper by individual members of the European Alzheimer's disease consortium [Bibr ED000182-bib-0002].

A major limitation of this meta‐analysis is the decision to pool together fundamentally different Aβ‐targeting monoclonal antibodies, including first‐generation compounds that all failed to significantly reduce amyloid burden, drugs that never demonstrated clinical efficacy, and only two agents currently approved for European clinical use. Such an approach risks diluting potential treatment effects and may inevitably lead to the conclusion that this therapeutic class is clinically ineffective.

Firstly, the authors pooled all Aβ‐targeting monoclonal antibodies, including both first and second generation. As the authors acknowledged in the introduction, all first generation Aβ‐targeting monoclonal antibodies failed to demonstrate a clinical benefit while mixed results were present in the second generation antibodies. A potential explanation for these mixed results is the different targeting, for instance Lecanemab targets soluble Aβ protofibrils while Donanemab targets Aβ plaques [Bibr ED000182-bib-0003 ED000182-bib-0004]. Therefore, pooling all antibodies does not seem correct and may mask significant clinical effects if only a limited number of antibodies (by targeting the right part of the amyloid pathway) are successful.

Secondly, significant clinical effects are expected after a long follow‐up period as the downstream effects after amyloid clearance take time. Only 6 out of the 17 included studies in this meta‐analysis, had a follow‐up period longer than 18 months, so follow‐up may be too short to demonstrate any significant clinical effect. Thirdly, the authors conclude that successful removal of amyloid from the brain does not seem to be associated with clinically meaningful effects, but all first‐generation compounds included in the analysis did not remove amyloid based on amyloid PET imaging. Further, no subgroup analysis between first and second generation compounds were performed and the authors did not investigate whether including only individuals with a significant decrease in cerebral amyloid load changed their findings. For instance in the TRAILBLAZER phase 2 trial, even after 76 weeks of therapy 20% of patients did not reach amyloid clearance and in this subset of patients no clinical effects are expected [Bibr ED000182-bib-0004]. In a systematic review of anti‐amyloid trials the degree of amyloid beta plaque load removal measured by amyloid PET was found to be associated with a slower clinical deterioration measured by the reduction of CDR‐SB, with CLARITY AD and TRAILBLAZER‐ALZ2 in the lowest range of this correlation [Bibr ED000182-bib-0005]. Therefore, the clinical benefit can be related to the degree of amyloid plaque removal. This correlation suggests that the amyloid pathway is a relevant target for AD, although a clinical meaningful effect (measured for instance by a 1‐point change on CDR‐SB or a 2‐point change on ADAS‐Cog 11 or 13) might require a longer trial period or longer clinical follow‐up.

Finally, sub‐group analysis with patient stratification based on tau load was not performed. This analysis would be relevant considering that Aβ‐targeting monoclonal antibodies are thought to be most successful in individuals with low to medium tau load.

In addition, emerging real‐world evidence from cohorts treated with the two currently approved anti‐amyloid monoclonal antibodies, lecanemab and donanemab, suggests that amyloid‐related imaging abnormalities (ARIA) may be less frequent and clinically more manageable than initially feared based on randomized clinical trials [Bibr ED000182-bib-0006 ED000182-bib-0007 ED000182-bib-0008 ED000182-bib-0009 ED000182-bib-0010 ED000182-bib-0011]. Across published real‐world cohorts, the overall incidence of ARIA has generally ranged between approximately 7% and 22%, while symptomatic ARIA has remained relatively uncommon, typically occurring in about 1–6% of treated patients. Reported ARIA‐E rates ranged from approximately 2% to 15%, whereas ARIA‐H rates were generally between 7% and 11% in studies reporting these outcomes separately. Importantly, most ARIA events were mild or asymptomatic and detected during routine MRI surveillance rather than following clinical deterioration. Severe complications, including macrohemorrhage, permanent neurological sequelae, or treatment‐related deaths, appear to be rare in routine clinical practice and have not been reported in the major real‐world cohorts published to date [Bibr ED000182-bib-0006 ED000182-bib-0007 ED000182-bib-0008 ED000182-bib-0009 ED000182-bib-0010 ED000182-bib-0011]. Furthermore, risk factors of ARIA (Apoe4 carriers, load of amyloid plaques and occipital localization of amyloid plaques (both evaluated by amyloid PET)) are known and can help to select patients having the most chance to benefit from anti‐amyloid therapy [Bibr ED000182-bib-0012]. Also, clinically significant ARIA cases were generally manageable with temporary treatment interruption and standard medical therapy, including corticosteroids when required. Notably, available real‐world studies also suggest that ARIA occurrence did not significantly influence the trajectory of cognitive change during follow‐up [Bibr ED000182-bib-0010]. Overall, currently available real‐world data support the view that, within experienced multidisciplinary centers, ARIA represents a clinically manageable safety event rather than an unpredictable or prohibitive treatment complication.

All together, the strong conclusions proposed by the authors should be interpreted with caution, as several methodological limitations may substantially influence the results. Future systematic reviews should consider differences in amyloid‐targeting mechanisms, degree of amyloid clearance, treatment duration, and biological disease stage before concluding that Aβ‐targeting monoclonal antibodies lack clinically meaningful effects.

## Reply: Amyloid‐beta‐targeting monoclonal antibodies for people with mild cognitive impairment or mild dementia due to Alzheimer’s disease

### Declarations of interest

***ER****and* ***FN****declare non‐financial interests, being unpaid consultants of the European Medicine Agency's Scientific Advisory Group Neurology (SAG‐N).* ***FN, EB, LV****, and* ***LS****declare non‐financial interests by practising as neurologists within a public institution.* ***ER, FN, SM****,* ***CB, CDS, LS, EB, LV, GF, CDG****, and* ***MGL****declare no commercial or non‐commercial conflicts of interest relevant to this review.* ***GF****and* ***FN****are joint Co‐ordinating Editors of Cochrane Multiple Sclerosis and Rare Diseases of the CNS. They are Senior Editors of the Cochrane Database of Systematic Reviews. They were not involved in the editorial process of the review.*

We thank Van Weehaeghe and colleagues for their commentary on our review [Bibr ED000182-bib-0013]. An important point the authors make is about pooling of studies using different monoclonal antibodies (mAb) against amyloid‐beta. In addressing these comments elsewhere we have highlighted that some of the earlier antibodies, including gantenerumab and aducanumab, removed amyloid from the brain [Bibr ED000182-bib-0014 ED000182-bib-0015].

Our findings demonstrate that the average treatment effect is smaller than established thresholds for cognitive function and dementia severity. In presenting separate meta‐analyses for each of the seven mAbs, we have shown that the average treatment effect for each mAb is also below the thresholds. These results support our overall conclusion that these drugs have no clinically meaningful effect based on the available evidence from phase III RCTs.

We agree that long‐term effects are unknown due to the limited study duration. We regret that the studies were not designed to be longer, in spite of widely available knowledge that in these study populations, 18 months may be too short to detect clinically meaningful effects on cognitive decline. We would like to stress that observational data, whether from open‐label extension studies or so‐called real‐world data, lack a control group, are prone to several sources of bias, and are not suitable to establish treatment effects, particularly when results from available phase III RCTs are not uniformly convincing.

The correlation between the extent of amyloid removal and clinical symptoms, as suggested by the authors, is not supported by strong evidence, as illustrated by a meta‐analysis including 14 randomised controlled studies on amyloid‐targeting drugs, showing no clear relation between amyloid‐beta removal and clinically detectable effects on cognition [Bibr ED000182-bib-0016]. We question the significance of any observable differences in amyloid removal if it is associated with a lack of clinically meaningful differences in cognitive function.

We note reports of an apparently lower rate of ARIA with use of mAb in clinical practice. Unfortunately, as explained in our systematic review, ARIA was generally reported in a poor and inconsistent way. Although these reports of lower incidence are encouraging, they are not well understood with respect to adverse events. If results on ARIA from real‐world data were to be evaluated, this should be done in a prospective and systematic way. Observational studies should consider adherence to surveillance protocols, feasibility of regular clinic attendance and take all potential sources of bias and confounding associated with observational studies into account.

Results from subgroup analysis, for instance stratified by level of tau deposition as suggested by the authors, can provide important hypothesis‐generating insights. If plausible, results could lead to a new, properly powered RCT to test such a hypothesis and confirm this is a reproducible finding.

The authors suggest that a new meta‐analysis, including the studies with longer follow‐up, is needed. We have analysed data from all the available endpoints from all the eligible studies, including those with longer intervention and follow‐up. Further pursuing meta‐analysis with the currently available studies will likely not lead to different conclusions.

Finally, a rigorous, objective synthesis of the best available evidence, developed by independent researchers free of conflict of interest is of vital importance to inform clinical decision making.

### Authors

**Francesco Nonino**
  Unit of Epidemiology and Statistics, IRCCS Istituto delle Scienze Neurologiche di Bologna, Bologna, Italy, f.nonino@ausl.bologna.it
**Silvia Minozzi**
  Laboratory of Methodology of Systematic Reviews and Guidelines production, Department of Experimental Oncology, Istituto di Ricerche Farmacologiche Mario Negri IRCCS, Milan, Italy
**Luisa Sambati**
  UOC Clinica Neurologica Rete Metropolitana (NeuroMet), IRCCS Istituto delle Scienze Neurologiche di Bologna, Bologna, Italy
**Cinzia Del Giovane**
  Department of Medical and Surgical Sciences for Children and Adults, University of Modena and Reggio Emilia, Modena, Italy
  Institute of Primary Health Care (BIHAM), University of Bern, Bern, Switzerland
**Elisa Baldin**
  Unit of Epidemiology and Statistics, IRCCS Istituto delle Scienze Neurologiche di Bologna, Bologna, Italy
**Maria Chiara Bassi**
  Medical Library, Azienda USL ‐ IRCCS di Reggio Emilia, Reggio Emilia, Italy
**Claudia De Santis**
  Unit of Epidemiology and Statistics, IRCCS Istituto delle Scienze Neurologiche di Bologna, Bologna, Italy
**Marien Gonzalez‐Lorenzo**
  Laboratory of Methodology of Systematic Reviews and Guidelines production, Department of Experimental Oncology, Istituto di Ricerche Farmacologiche Mario Negri IRCCS, Milan, Italy
**Luca Vignatelli**
  Unit of Epidemiology and Statistics, IRCCS Istituto delle Scienze Neurologiche di Bologna, Bologna, Italy
**Graziella Filippini**
  Independent Researcher, Milan, Italy
**Edo Richard**
  Department of Neurology, Radboud University Medical Centre, Nijmegen, Netherlands
  Department of Public & Occupational Health, Amsterdam University Medical Centre, University of Amsterdam, Amsterdam, Netherlands
